# The Translation, Cross-Cultural Adaptation and Validation of the Italian Version of the Hip and Groin Outcome Score Questionnaire for a Young and Active Population

**DOI:** 10.3390/healthcare12171755

**Published:** 2024-09-03

**Authors:** Andrea Ricci, Alex Rossi, Mirko Zitti

**Affiliations:** 1 Department of Health Sciences, Alma Mater Europaea–European Center of Maribor, 2000 Maribor, Slovenia; andrea.ricci@almamater.si; 2 Department of Clinical Sciences and Translational Medicine, University of Rome Tor Vergata, 00133 Rome, Italy; alex.rossi@uniroma2.it; 3 Department of Human Neuroscience, University of Rome “La Sapienza”, 00185 Rome, Italy

**Keywords:** hip and groin outcome score, groin pain, hip pain, cross-cultural validation, Italian validation

## Abstract

The HAGOS (Hip and Groin Outcome Score) questionnaire is a valid and reliable measure of the self-assessment of symptoms, activity limitation, participation restriction, and quality of life (Qol) of subjects with hip and/or groin pain. The aims of this study are to translate and transculturally adapt the HAGOS into Italian (HAGOS-I) and to assess its internal consistency, validity, and reliability in physically active, young, and middle-aged subjects. The translation and transcultural adaptation of (HAGOS-I) was carried out according to international guidelines. Eight-one subjects (mean age 28.19) were included in this study. All the participants completed the HAGOS-I, the Lower Extremity Functional Scale (LEFS-I), the Oxford Hip Score (OHS-I), and the Short Form 36 Health Surveys (SF-36-I). The Cronbach’s α for the six HAGOS subscales ranged from 0.63 to 0.87. Statistically significant correlations were obtained between the six HAGOS-I subscales and the LEFS-I (rs = 0.44–0.68; *p* < 0.01). Only one HAGOS-I subscale (Participation in Physical Activities) did not reach statistical significance with the OHS-I, while the remaining five had a moderate correlation (rs = 0.40–0.60; *p* < 0.01). The test–retest reliability (Intraclass Correlation Coefficient) ranged from 0.57 to 0.86 for the six HAGOS-I subscales. The HAGOS-I is a valid and reliable instrument that can be used in clinical settings with young and middle-aged subjects with hip and/or groin pathologies.

## 1. Introduction

Hip and/or groin injuries are more common in sports such as football, ice hockey and rugby, but can occur in most sports with elements of running, changing direction and jumping. They account for 5% to 18% of all sport-related injuries [1,2,3].

In men’s football, a seven-year prospective study of elite players found hip and groin injuries to account for 12% to 16% of all injuries. In total, the incidence was 1.1 injuries/1000 training h, with matches contributing 3.5 injuries/1000 h. Injuries related to the adductor muscle complex were by far the most common, followed by injuries related to the iliopsoas muscle. A study of sub-elite football players showed the same epidemiological pattern with a higher prevalence of adductor-related injuries, followed by iliopsoas- and abdominal-related injuries. The incidence was lower at 0.4 injuries/1000 h total training time [4,5].

Pain in the hip and groin regions is a common musculoskeletal disorder in young and middle-aged populations that affects physical function and health-related quality of life [6]. This disorder can be a long-term condition, as full recovery is difficult [7,8], as well as having a large financial impact on society in terms of absence from work and disability [9].

There is a general consensus that Patient-Reported Outcomes (PROs) should be identified as the gold standard in the assessment of musculoskeletal conditions, especially for what the patient perceives in relation to quality of life [10,11,12]. Although their usefulness is widely accepted, to date there is a lack of valid, reliable and responsive PRO questionnaires in the Italian language for young, physically active patients with persistent hip and/or groin pain [13].

In 2010, the Consensus-Based Standards for the Selection of Health Status Measurement Instruments (COSMINs) published a checklist that should be used to develop and evaluate health-related PROs (HR-PROs) [14]. The checklist was designed to be used as a guide in the development of HR-PROs and to assess the quality of studies by measuring the properties of HR-PROs.

The HAGOS (Copenhagen Hip and Groin Outcome Score) is a pathology-specific questionnaire for subjects suffering from hip and/or groin disorders published in 2011 by Thorborg et al. and validated according to the COSMIN checklist [15]. The HAGOS consists of 37 items divided into six subscales that assess Symptoms, Pain, Physical Functionality in Activities of Daily Living, Physical Functionality in Sport and Leisure Time, Participation in Physical Activities, and quality of life in relation to the hip and/or groin area. All the items are scored on a 5-point Likert scale from 0 (no symptoms) to 4 (extreme symptoms). The overall scores for the individual subscales are calculated by summing the scores of the individual items, with a higher final total score indicating better functionality.

This instrument has already been used as an endpoint in some research [16,17,18] and has already been validated in English, Swedish and Dutch [15,19,20].

Currently, two transcultural translation works have been proposed in the Italian language by Negrau et al. [21] and Bisciotti et al. [22], which differ in some aspects from the original work [15] in terms of the population examined [21,22] and in the psychometric parameters considered [22].

The aim of this study is, therefore, to transculturally adapt and validate the Italian version of the HAGOS by administering it to a young and active population in order to analyse a population sample that has not yet been studied, but is similar to the original study.

## 2. Materials and Methods

### 2.1. Translation Procedure and Cultural Adaptation

The HAGOS was translated into Italian according to the guidelines proposed by Beaton et al. [23]. Initially, two native Italian translators (A.R. and M.M.) translated the HAGOS questionnaire from English into Italian. The two translators, together with an expert committee (3 physiotherapists specialised in orthopaedic manual therapy (AL.R., S.M., and M.Z.) and 1 physiotherapist specialised in sports physiotherapy (R.N)), synthesised the two translations into one. Subsequently, two native English speakers fluent in Italian (A.P. and B.B.C.) performed independent back-translation (from Italian to English). Any inconsistencies between the original scale and the backward translation were resolved by the committee of experts and the translators involved; the pre-final version of the Italian HAGOS was thus constructed (HAGOS-I). The synthesis process was carefully documented, and differences were resolved by consensus. The pre-final version of the HAGOS-I was tested in a pilot test to assess acceptability, consisting of administering it to 10 subjects who did complain of hip and/or groin pain. They were encouraged to comment on the comprehension of the items. This pilot test was carried out to ensure that the questions were not interpreted as intrusive, as well as to make sure that subjects with no health literacy could understand the questions. The end result is the final version of the HAGOS-I (see Supplementary Materials).

### 2.2. Participants and Procedures

Participants were recruited from 3 physiotherapy clinics specializing in sports and musculoskeletal rehabilitation (3 in Northern Italy and 1 in Central Italy).

**Figure 1:**
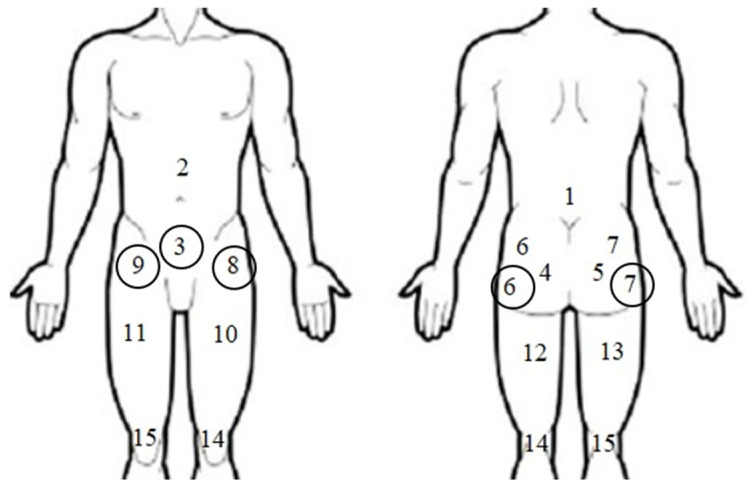
This pain drawing shows the areas of body pain taken into account (black circle) as criteria for the inclusion of subjects in this study.

The inclusion criteria were sent to the physiotherapists of these clinics. The inclusion criteria were as follows: (1) age 18–45 years, and period of diagnosis 2017–2022; (2) subjects who required treatment for their hip and/or groin pain; (3) subjects who were limited in their activities due to hip and/or groin pain; (4) subjects who had hip and/or groin pain in the 14 days preceding the date of administration of the questionnaire; (5) subjects who had hip and/or groin pain of more than 6 weeks duration (pain of more than 6 weeks duration was previously defined as persistent with reference to the population under investigation [24]); (6) subjects who had pain in the hip and/or groin area located in one of the five predefined regions on a pain body zone drawing (region 3, 6, 7, 8 or 9, see Figure 1); and (7) subjects who were physically active for at least 2.5 h per week.

If the specialist (physiotherapist specialising in orthopaedic manual therapy or sports physiotherapy) suspected that the pain in the hip and/or groin area was not of musculoskeletal origin, or if the patients had self-reported limiting comorbidities, this was grounds for exclusion from this study [25].

The patients included fell into one of the five categories established by the Doha meeting [26], in which the correct terminology for defining groin pain in athletes was established as follows: adductor-related groin pain, ileopsoas-related groin pain, Inguinal-related groin pain, pubic-related groin pain, or hip-related groin pain.

The patients were contacted by e-mail and informed about the purpose of research by the staff responsible for the study, and written informed consent was obtained from those who agreed to participate.

The patients completed questionnaires using Google Sheets forms (©), and the data were collected via the Google Drive platform (©).

### 2.3. Validity

Construct validity is the degree to which scores on a PRO instrument are consistent with a priori assumptions based on the assumption that the instrument validly measures the construct being measured [27].

Construct validity was studied by correlating the subscale scores of the HAGOS with the Lower Extremity Functional Scale (LEFS) [28], the Oxford Hip Score (OHS) [29] and the Short Form 36 scale (SF-36) [30].

The LEFS is a functional status questionnaire consisting of 20 items applicable to a broad spectrum of patients with pathological conditions of musculoskeletal origin in the lower extremities. The items investigate the degree of difficulty in performing different physical activities due to lower limb problems. It was validated by Cacchio et al. in 2010 (LEFS-I) [28].

The OHS is a questionnaire with good measurement properties in the context of outcome assessment in primary or revision hip replacement. The questionnaire consists of 12 items assessing pain and hip function. It was validated in Italian by Martinelli et al. in 2011 (OHS-I) [29].

The SF-36 is a questionnaire consisting of 36 questions about the general status of patients. It consists of eight health-related subscales (physical function, physical role, physical pain, general health, vitality, social function, emotional role, and mental health), which are then aggregated into two main scores. The official Italian version was created by Apolone et al. (1997, IQOLA project) [30].

Spearman’s correlation coefficients were calculated between the subscales of the HAGOS-I and the subscales of the SF-36-I, the LEFS-I and the OHS-I. Spearman’s correlation coefficients were interpreted by Cohen as follows: <0.30 = small; 0.30–0.50 = moderate; and >0.50 = large [31].

Since the HAGOS was designed to measure physical functionality rather than mental and/or social functionality, the highest correlations were expected between the subscales of the HAGOS and the subscales of the SF-36-I, the LEFS-I and the OHS-I that are supposed to measure physical functionality (convergent validity) [32].

The highest correlations were expected between the subscales of the HAGOS and the subscales of the SF-36-I, i.e., physical functionality (PF), Role Limitations due to Physical Health Problems, body pain (BP), the LEFS-I and the OHS-I (convergent validity). Fewer correlations were expected between the subscales of the HAGOS-I and the subscales of the SF-36-I (Perception of General Health; Vitality, Social Functioning; Role Limitations due to Emotional Problems; and General Mental Health) that are supposed to measure mental and/or social functioning (divergent validity).

The a priori assumptions were that the correlation between the subscales of the HAGOS-I “Sport/Sport and Recreation function”(Sport/Rec) and the subscale “Participation in Physical Activity” (PA) of the SF-36-I must be at least 0.5; the correlation between the subscale “Bodily pain” of the SF 36-I and the HAGOS-I subscales “Pain” and “Symptoms” are at least 0.5; and a correlation between 0.3 and 0.5 was hypothesised for the subscales “Symptoms” of the HAGOS-I and “OHS-I”.

A strong correlation was assumed between the HAGOS-I subscales PA and “Sport/Ric” and the LEFS-I scale.

If 75% or more of the arbitrarily set number of hypotheses were confirmed, the construct validity of the HAGOS-I would be considered good [33].

### 2.4. Internal Consistency

Internal consistency is the degree of correlation between items [33]. Internal consistency was measured for the 6 subscales of the HAGOS-I from the values at the baseline and was considered “good” if the value of Cronbach’s alpha was between 0.70 and 0.95 [34].

### 2.5. Reliability

Test–retest reliability is defined as the proportion of total variance in the measurements due to true differences between patients [34]. The intraclass 2-way mixed model (ICC) correlation coefficient was calculated using the statistical programme SPSS software version 28.0 (©) for each of the six HAGOS-I scales. Test–retest reliability was assessed after 1–3 weeks. This time interval between the test and the retest was chosen following the original validation of the HAGOS, in which it was thought to be an interval long enough to avoid the recall of the previous responses and short enough so that the condition in most cases did not change. At the retest, the subjects had to state whether their pathological condition in the hip and/or groin area had ‘improved’, ‘unchanged’ or ‘worsened’ since the initial test. The patients who reported an ‘unchanged’ condition were considered stable and included in test–retest reliability analysis [15,34].

The CCI is a value between 0 and 1, where a value below 0.50 indicates poor reliability, between 0.50 and 0.75 indicates moderate reliability, between 0.75 and 0.90 indicates good reliability, and above 0.90 indicates excellent reliability [33].

### 2.6. Measurement Error

Measurement error is the systematic and random error of the score, which is not attributed to the construct being measured [34]. Measurement error is expressed as the standard error of the mean (SEM) using the formula SD × √1-ICC, with SD being the mean of the standard deviations between the two measurements (test and retest) [32]. The minimal detectable change (MDC), the change in a score that exceeds the measurement error, was calculated at the individual level as ESM × 1.96 × √2 and at the group level as ESM × 1.96 × √2/√n.

### 2.7. Ceiling and Floor Effects

The presence of floor and ceiling effects can influence the validity and reliability of an instrument. Floor and ceiling effects are defined as 15% of the patients reaching the minimum or maximum score, respectively [33].

### 2.8. Statistical Analysis

Descriptive statistics were used to report the patient demographics as the mean and standard deviation (SD). The Kolmogorov–Smirnov test was used to assess the normality hypothesis. At *p* < 0.05, it was considered statistically significant. The data were entered into a Microsoft Excel spreadsheet (Microsoft Corporation, Redmond WA (©)) and analysed using SPSS software version 28.0 (©).

## 3. Results

The final HAGOS version can be found in ‘Supplementary Materials’.

Forward and backward translations did not cause any major problems. The cross-cultural adaptation of the HAGOS revealed no major problems, and no discrepancies were found; the pilot testing ended without any reports from the participants.

A total of 81 patients completed all the questionnaires. The demographic data of the cohort are listed in Table 1. In Table 2 are the listed scores for each HAGOS subscale. Some missing data were found for the SF-36 scale, which were corrected following the guidelines of the respective manuals.

Statistical analysis (Table 3) showed a strong correlation between the sport subscale of the HAGOS-I and the PF-SF36 subscale (0.683) and the LEFS (0.634); a moderate correlation between the Pain subscale of the HAGOS-I and the BP-SF36 subscale (0.512); a strong correlation between the PA subscale of the HAGOS-I and the LEFS (0.681); and finally, a strong correlation between the Symptoms subscale of the HAGOS-I and the OHS (0.511).

For the construct validity of the HAGOS-I, Spearman correlation coefficients were calculated between the HAGOS-I and the SF-36-I, between the HAGOS-I and the LEFS-I, and between the HAGOS-I and the OHS-I (Table 4).

The internal consistency for all the subscales of the HAGOS-I ranged from a Cronbach’s alpha of 0.63 to 0.87 (Table 4).

The Intraclass Correlation Coefficient (ICC) ranged between 0.57 and 0.86 (Table 4).

The MDC for the six subscales varied between 7.40 and 33.84 at the individual level and between 1.37 and 6.28 at the group level (Table 4).

The floor and ceiling effects, set as present if more than 15% of the patients reported the worst (0) or highest (100) possible scores, were detected for the subscale PA at the baseline (Table 4).

## 4. Discussion

In this study, the HAGOS questionnaire was successfully translated and transculturally adapted into Italian. The translated version of the HAGOS (HAGOS-I) showed that the questionnaire is valid and reliable for assessing the health-related functional and quality-of-life statuses of individuals with hip and/or groin pain identified by Weir et al. in the 2015 Doha Consensus [26].

The distribution of anatomical lesions in the population of the subjects examined (physically active subjects experiencing groin pain for at least 2.5 h/week for at least 6 weeks) shows a difference to the characteristic prevalence already investigated in other previous studies [35]; in particular, a high percentage of subjects complaining of pain in the hip and groin areas compared to the involvement of the psoas and the abdominal area is noted. This may probably be due to the multiplicity of physical activities practised by the subjects recruited in this study compared to those in Holmich et al.’s study [1], which examined a population of footballers.

The internal consistency coefficients were high, ranging between 0.63 and 0.87 for all the subscales. Compared to the five subscales (Pain, ADL, Sport/Recreational Activities, Participation in Physical Activities, and quality of life), which have excellent internal consistency values (between 0.8 and 0.87), the “Symptoms” subscale has a lower, but still good correlation value (0.63), probably due to the fact that the retest subjects continued with their rehabilitation treatment, leading to a variation in the symptoms reported.

These data are in line with the original validation published by Thorborg et al. [15], Thomeè et al. (Swedish validation) [20], and Brans et al. (Dutch validation) [19].

In line with the original validation study [15], a few patients reported a minimum or maximum score (floor/ceiling effect) for the HAGOS, indicating the possibility of measuring both improvement and worsening over time. Similarly, for the subscale PA, no. 13 has the highest number of subjects with minimum scores. This is due to the fact that the subscale provides a minimum score (0) for non-participation in the patient’s preferred physical activities (“NEVER” item no. 5–PA1) which is very accurate in describing the patient’s condition, and there are no further items that could explain further worsening.

The construct validity of the HAGOS-I was obtained by comparing the subscales of the HAGOS-I with the SF-36-I, the LEFS-I and the OHS-I. The a priori assumptions made were fully met, indicating good construct validity [33]. The a priori assumptions were as follows: The correlation between the subscales of the HAGOS-I “Sport/Ric” and the subscale “PF” of the SF-36-I was at least 0.5. The correlation between the subscale “BP” of the SF 36-I and the subscales HAGOS-I “Pain” and “Symptoms” was at least 0.5 (satisfied with subscale “Pain” [0.51], while not satisfied with subscale “Symptoms” [0.34]). The correlation was assumed between 0.3 and 0.5 for the subscale “Symptoms” of the HAGOS-I and “OHS-I”, where the value is 0.51. A strong correlation was assumed between the HAGOS-I “PA” and “Sport/Ric” subscales and the LEFS-I scale, respectively, 0.681 and 0.639. If 75% or more of the arbitrarily set number of hypotheses were confirmed, the construct validity of the HAGOS-I would be considered good [33].

The reliability of the HAGOS-I is good; it was above 0.7 for all the subscales except for the subscale “Symptoms”, which has a moderate reliability value (0.57). The latter value could be justified by the sample size at retest (n.29), the variability in the presentation of persistent groin pain [36], and the fact that the patients were undergoing physiotherapy treatment to improve their clinical situation.

Overall, the SEM values are not comparable with those in the original validation [15]; the large MDC values at the individual level (individual MCD) in the present study were common to the patient-reported questionnaires, refs. [37,38], indicating that patient-reported questionnaires may be problematic for use at the individual level due to their inability to detect minimal, but still clinically important changes. The low MDC values at the group level (between 1.37 and 6.28) indicate that the HAGOS-I is highly usable in inter-group comparisons.

Ultimately, the present work complements and differs from the previously published validation in some aspects. The other existing validations focused on specific populations such as football players [22] and patients undergoing hip replacement due to various conditions (hip dysplasia, osteonecrosis, femoral neck fracture, and hip arthrosis) [21]. In contrast, our study closely aligns with the original sample, which included young-to-middle-aged, physically active patients with hip and/or groin pain who were physically active for at least 2.5 h/week who met the original inclusion/exclusion criteria. The only difference is that our sample ranged from ages 18 to 45, whereas Thorborg et al.’s [15] original study included participants aged 18–63.

The other differences are particularly evident when compared to the study by Bisciotti et al. [22]. Our study included analyses that were not present in their validation, such as the calculation of the SEM, the assessment of ceiling and floor effects, and a clear and transparent description of the test–retest timing. Additionally, unlike Bisciotti’s study, which included only male participants, our study also included female patients, in line with the original validation. Another limitation of this study is that it is not indexed in databases.

From the results, it can be deduced that this validation can be regarded as the first study that has been successfully conducted on young and middle-aged (18–45 years) subjects with groin pain for at least 6 weeks, as stated in the original study by Thorborg et al. [15].

### Limitations of the Study

Compared with the original validation by Thorborg et al. [15], the limitations of the study concern the lack of MIC assessment and the measurement of responsiveness, which requires medium-term follow-up, and the reference age investigated. A larger and more heterogeneous sample, both in terms of age and clinical conditions, could have provided more generalizable results.

In addition, the patients included in the study were receiving physical therapy to improve their clinical condition. This variable could have influenced their pain responses and perceptions of functionality.

## 5. Conclusions

The HAGOS was successfully translated transculturally into Italian (HAGOS-I). This study demonstrated that the HAGOS-I is a valid and reliable tool; therefore, this scale can help Italian clinicians in the evaluation of the functional status and health-related quality of life of young (18–45 years) and physically active patients with hip and/or groin pain for at least 6 weeks.

## Figures and Tables

**Table 1:** Descriptive data for patients in validity study and test–retest-reliability study.

	Baseline Data Patients	Test–Retest Reliability Study (Media) [dev. Standard]
Patient	81 (66m, 15f)	29 (25m, 4f)
Age	(28.19) [6.17]	(27.97) [6.18]
Adductor-related GP	32 (39.5%)	13 (44.83%)
Ileopsoas-related GP	9 (11.10%)	4 (13.8%)
Inguinal-related GP	15 (18.50%)	4 (13.8%)
Pubic-related GP	5 (6.20%)	1 (3.43%)
Hip-related GP	20 (24.7%)	7 (24.14%)
>6 w symptoms	16 (19.75%)	3 (10.34%)
>12 w symptoms	12 (14.80%)	4 (13.8%)
>6 w symptoms	22 (27.15%)	9 (31%)
>12 w symptoms	31 (38.3%)	13 (44.86%)
Drugs (paracetamol and NSAIDs)	23 (28.4%)	10 (34.49%)
Drugs(opioids)	1 (1.2%)	1 (3.43%)
No drugs	57 (70.4%)	18 (62.08%)
PA > 2.5 h/week	29 (35.80%)	13 (44.86%)
PA > 5 h/week	34 (42%)	9 (31%)
PA > 10 h/week	18 (22.20%)	7 (24.14%)

GP: groin pain; PA: physical activity; w: week; NSAIDs: non-steroidal anti-inflammatory drugs.

**Table 2:** HAGOS subscales validity study, test reliability study, and retest reliability study.

	Validity Study (Media) [dev. Standard]	Test Reliability Study (Media) [dev. Standard]
Patients	81	29
HAGOS subscale Symptoms	62.08 (13.36)	61.82 (13.77)
HAGOS subscale Pain	73.36 (14.64)	73.19 (16)
HAGOS subscale ADL	78.09 (16.69)	76.90 (18.77)
HAGOS subscale Sport/Rec	60.07 (18.53)	60.78 (20.60)
HAGOS subscale PA	45.22 (30.59)	55.60 (29.99)
HAGOS subscale Qol	46.05 (19.60)	48.28 (20.01)

ADL: activities daily life; Rec: recreation function; PA: Participation in Physical Activity; Qol: quality of life; HAGOS: Hip and Groin Outcome Score.

**Table 3:** Correlation between HAGOS scores and other outcome measures.

	HAGOS S	HAGOS P	HAGOS PA	HAGOS Sport/Rec
SF36 PF	0.410 *	0.599 *	0.598 *	0.683 *
	<0.001	<0.001	<0.001	<0.001
SF36 BP	0.340 *	0.512 *	0.395 *	0.545 *
	0.002	<0.001	<0.001	<0.001
OHS	0.511 *	0.606 *	0.518 *	0.462 *
	<0.001	<0.001	<0.001	<0.001
LEFS	0.447 *	0.658 *	0.681 *	0.639 *

* Stat. Significant *p* < 0.05. SF36: Short Form Health Surveys scale; OHS: Oxford Hip Score; LEFS Low: Extremity Functional Scale; PF: Physical Function; BP: Bodily pain; S: Symptoms; P: Pain; PA: Participation in Physical Activity; Rec: recreation function; HAGOS: Hip and Groin Outcome Score.

**Table 4:** Descriptive statistics and test–retest reliability of HAGOS-I.

	Number of Items	Cronbach Alpha Values	ICC	CI ICC	SEM	MDC Individual	MDC Group	Floor Effect. (%) (n.81)	Ceiling Effects (%) (n.81)
HAGOS subscale Symptoms	7	0.63	0.57	(0.27–0.77)	8.96	24.83	4.61	0	0
HAGOS subscale Pain	10	0.82	0.83	(0.67–0.91)	2.67	7.40	1.37	0	2 (2.46)
HAGOS subscale ADL	5	0.80	0.84	(0.68–0.91)	7.19	19.93	3.70	0	8 (9.87)
HAGOS subscale Sport/Rec	8	0.87	0.84	(0.68–0.92)	8.72	24.17	4.49	1 (1.23)	0
HAGOS subscale PA	2	0.87	0.86	(0.72–0.93)	12.21	33.84	6.28	13 (16.05)	5 (6.17)
HAGOS subscale Qol	5	0.80	0.71	(0.45–0.86)	11.44	31.71	5.89	2 (2.46)	0

ADL: activities daily life; Rec: recreation function: PA: Participation in Physical Activity: Qol: quality of life; ICCL Intraclass Correlation Coefficient; CI: confidence interval; SEM: standard error measurement; MDC: minimal detectable change; HAGOS: Hip and Groin Outcome Score.

## Data Availability

The data supporting the results of this study are available by correspondence upon reasonable request.

## Supplementary Materials

The following supporting information can be downloaded at: https://www.mdpi.com/article/10.3390/healthcare12171755/s1.
