# Supplementary material for: The Translation, Cross-Cultural Adaptation and Validation of the Italian Version of the Hip and Groin Outcome Score Questionnaire for a Young and Active Population
Source: Healthcare (Basel). 2024 Sep 3;12(17):1755. doi: 10.3390/healthcare12171755 (PMC11394954; doi:10.3390/healthcare12171755)

HAGOS-I (Questionnaire and manual)

HAGOS-I

Questionario riguardante problematiche all’anca e/o alla zona inguinale

Data odierna _____/______/______ Data di Nascita _____/______/______

Nome: ____

ISTRUZIONI: Questo questionario richiede il tuo parere riguardo il tuo problema all'anca e/o zona inguinale. Dovresti rispondere alle domande considerando la funzionalità a livello dell'anca e/o della zona inguinale durante la **scorsa settimana**. Questa informazione ci aiuterà a tenere traccia di come ti senti, e quanto bene riesci a svolgere le tue attività abituali.

Rispondi ad **ogni** domanda spuntando la casella appropriata. Spunta solo una casella per ogni domanda. Se un quesito non ti riguarda o non ne hai avuto esperienza nella scorsa settimana per favore esprimi l'ipotesi migliore in modo che sia la più accurata possibile.

Sintomi

Le risposte a queste domande dovranno essere fornite considerando i **sintomi** all'anca e alla zona inguinale e le difficoltà durante **l'ultima settimana**.

S1 Senti fastidio all'anca e/o in zona inguinale?

Mai	Raramente	Qualche volta	Spesso	Sempre
☐	☐	☐	☐	☐

S2 Hai sentito dei click o altri tipi di rumore dall'anca e/o dalla zona inguinale?

Mai	Raramente	Qualche volta	Spesso	Per tutto il tempo
☐	☐	☐	☐	☐

S3 Hai difficoltà a fare uno stretching ampio in laterale delle gambe?

Nessuna	Poca	Moderata	Grave	Estrema
☐	☐	☐	☐	☐

S4 Hai difficoltà a fare passi lunghi quando cammini?

Nessuna	Poca	Moderata	Grave	Estrema
☐	☐	☐	☐	☐

S5 Hai provato sensazioni improvvise tipo fitta/pugnalata all'anca e/o in zona inguinale?

Mai	Raramente	Qualche volta	Spesso	Per tutto il tempo
☐	☐	☐	☐	☐

Rigidità

Le domande seguenti riguardano quanta rigidità hai riscontrato durante **l'ultima settimana** all'anca e/o in zona inguinale. La rigidità è una sensazione di restrizione o rallentamento nel modo in cui muovi l'anca e/o la zona inguinale.

S6 Quanto è grave la rigidità all'anca e/o in zona inguinale dopo il risveglio la mattina?

Nessuna	Lieve	Moderata	Grave	Estrema
☐	☐	☐	☐	☐

S7 Quanto è grave la rigidità all'anca e/o in zona inguinale dopo la posizione seduta, distesa o di riposo **a fine giornata**?

Nessuna Lieve Moderata Grave Estrema

☐ ☐ ☐ ☐ ☐

Dolore

P1 Quanto spesso hai avuto dolore all' anca e/o in zona inguinale?

Mai Mensilmente Settimanalmente Quotidianamente Sempre

☐ ☐ ☐ ☐ ☐

P2 Quante volte hai dolore in zone diverse dall'anca e/o dalla zona inguinale che pensi possano essere in relazione al problema dell'anca e/o della zona inguinale?

Mai Mensilmente Settimanalmente Quotidianamente Sempre

☐ ☐ ☐ ☐ ☐

Le seguenti domande riguardano quanto dolore hai provato durante la **scorsa settimana** all'anca e/o in zona inguinale. **Quanto dolore all'anca e/o in zona inguinale hai provato durante le seguenti attività?**

P3 Estendere completamente l'anca

Nessuno Lieve Moderato Grave Estremo

☐ ☐ ☐ ☐ ☐

P4 Flettere completamente l'anca

Nessuno Lieve Moderato Grave Estremo

☐ ☐ ☐ ☐ ☐

P5 Camminare su e giù per le scale

Nessuno Lieve Moderato Grave Estremo

☐ ☐ ☐ ☐ ☐

P6 Di notte mentre sei nel letto (dolore che disturba il sonno)

Nessuno Lieve Moderato Grave Estremo

☐ ☐ ☐ ☐ ☐

P7 In posizione seduta o distesa

Nessuno Lieve Moderato Grave Estremo

☐ ☐ ☐ ☐ ☐

Le seguenti domande riguardano quanto dolore hai provato durante la **scorsa settimana** all'anca e/o in zona inguinale. **Quanto dolore hai provato all'anca e/o in zona inguinale durante le seguenti attività?**

P8 In piedi

Nessuno Lieve Moderato Grave Estremo

☐ ☐ ☐ ☐ ☐

P9 Camminando su una superficie dura (asfalto, cemento, ecc)

Nessuno Lieve Moderato Grave Estremo

☐ ☐ ☐ ☐ ☐

P10 Camminando su una superficie irregolare

Nessuno Lieve Moderato Grave Estremo

☐ ☐ ☐ ☐ ☐

Funzionalità fisica, vita quotidiana

Le seguenti domande riguardano la tua funzionalità fisica**. Per ognuna delle seguenti attività per favore indica il grado di difficoltà che hai provato nella scorsa settimana dovuto alla tua problematica all'anca e/o in zona inguinale.**

A1 Salendo le scale

Nessuna Lieve Moderata Grave Estrema

☐ ☐ ☐ ☐ ☐

A2 Piegandoti in avanti, ad esempio per prendere qualcosa dal pavimento

Nessuna Lieve Moderata Grave Estrema

☐ ☐ ☐ ☐ ☐

A3 Entrando/uscendo dall'automobile

Nessuna Lieve Moderata Grave Estrema

☐ ☐ ☐ ☐ ☐

A4 Rimanendo disteso nel letto (girarsi o mantenere la stessa posizione dell'anca per un tempo prolungato)

Nessuna Lieve Moderata Grave Estrema

☐ ☐ ☐ ☐ ☐

A5 Svolgendo mansioni domestiche pesanti (lavare i pavimenti, passare l'aspirapolvere, spostare pacchi pesanti ecc.)

Nessuna Lieve Moderata Grave Estrema

☐ ☐ ☐ ☐ ☐

Funzionalità, sport e attività ricreative

Le seguenti domande riguardano la tua funzionalità fisica quando partecipi ad attività di alto livello. Rispondi ad **ogni** domanda spuntando la casella appropriata. Se un quesito non ti riguarda o non ne hai avuto esperienza nella scorsa settimana per favore esprimi la "migliore ipotesi" in modo che la risposta sia la più accurata possibile. **Dovresti rispondere alle domande considerando il grado di difficoltà che hai provato durante le seguenti attività nella scorsa settimana dovute alle tue problematiche all' anca e/o in zona inguinale.**

SP1 Accovacciarsi

Nessuna Lieve Moderata Grave Estrema

☐ ☐ ☐ ☐ ☐

SP2 Correre

Nessuna Lieve Moderata Grave Estrema

☐ ☐ ☐ ☐ ☐

SP3 Ruotare/fare da perno su di un arto inferiore in carico

Nessuna Lieve Moderata Grave Estrema

☐ ☐ ☐ ☐ ☐

SP4 Camminare su una superficie irregolare

Nessuna Lieve Moderata Grave Estrema

☐ ☐ ☐ ☐ ☐

SP5 Correre più forte che puoi

Nessuna Lieve Moderata Grave Estrema

☐ ☐ ☐ ☐ ☐

SP6 Portare la gamba con forza avanti e/o di lato, come nel calciare, pattinare, ecc

Nessuna Lieve Moderata Grave Estrema

☐ ☐ ☐ ☐ ☐

SP7 Movimenti improvvisi esplosivi che coinvolgono veloci giochi di piedi, come accelerazioni, decelerazioni, cambi di direzione, ecc.

Nessuna Lieve Moderata Grave Estrema

☐ ☐ ☐ ☐ ☐

SP8 Situazioni dove l'arto inferiore è allungato verso una posizione esterna al corpo (come quando l'arto è posizionato il più lontano possibile dal corpo)

Nessuna Lieve Moderata Grave Estrema

☐ ☐ ☐ ☐ ☐

Partecipazione alle attività fisiche

Le seguenti domande riguardano la tua capacità di partecipare alle tue attività fisiche preferite. Le attività fisiche includono le attività sportive così come tutte le altre forme di attività in cui sei leggermente in affanno. **Quando rispondi a queste domande considera in che grado la tua capacità di partecipare ad attività fisiche durante la scorsa settimana è stata influenzata dalla problematica all'anca e/o alla zona inguinale.**

PA1 Sei stato in grado di partecipare alle tue attività fisiche preferite fino a quando hai voluto?

Sempre Spesso Qualche volta Raramente Mai

☐ ☐ ☐ ☐ ☐

PA2 Sei stato in grado di partecipare alle tue attività fisiche preferite al tuo livello di performance normale?

Sempre Spesso Qualche volta Raramente Mai

☐ ☐ ☐ ☐ ☐

Qualità di vita

Q1 Quanto spesso sei preoccupato del tuo problema all'anca e/o in zona inguinale?

Mai Mensilmente Settimanalmente Quotidianamente Costantemente

☐ ☐ ☐ ☐ ☐

Q2 Hai modificato il tuo stile di vita evitando attività potenzialmente dannose all'anca e/o alla zona inguinale?

Affatto Di poco Moderatamente Severamente Totalmente

☐ ☐ ☐ ☐ ☐

Q3 In generale, quanta difficoltà hai con l'anca e/o la zona inguinale?

Nessuna Lieve Moderata Grave Estrema

☐ ☐ ☐ ☐ ☐

Q4 La tua problematica all'anca e/o alla zona inguinale colpisce il tuo umore in modo negativo?

Affatto Raramente Qualche volta Spesso Sempre

☐ ☐ ☐ ☐ ☐

Q5 Ti senti limitato dal tuo problema all'anca e/o alla zona inguinale?

Affatto Raramente Qualche volta Spesso Sempre

☐ ☐ ☐ ☐ ☐

Molte grazie per il completamento di tutte le domande di questo questionario

Una guida per l'utente del questionario HAGOS

HAGOS: HAGOS è una misura di outcome riferita dal paziente che impiega delle scale Likert a cinque voci. IL questionario HAGOS copre 6 dimensioni (sottoscale): sintomi, dolore, funzione nella vita quotidiana (ADL), Funzione nello sport e attività ricreative (Sport/Rec), Partecipazione alle Attività Fisiche (PA) e qualità della vita correlata all'anca e/o all'inguine (QOL).

Dati mancanti: se una X viene posizionata all'esterno di una casella, viene utilizzata la casella più vicina. Se ci sono due caselle contrassegnate, si sceglie la casella che indica i problemi più gravi. I dati mancanti sono trattati come tali; uno o due valori mancanti sono sostituiti con il valore medio per dimensione. Se più di due elementi sono omessi per le sottoscale Sintomi, Dolore, ADL, Sport/Rec e QOL, la risposta è considerata non valida. Se viene omesso più di 1 elemento per la sottoscala PA, la risposta è considerata non valida.

Calcolo del punteggio: le 6 sottoscale di HAGOS vengono valutate separatamente: Sintomi (7 elementi); Dolore (10 elementi); ADL (5 elementi); Sport/Rec (8 elementi); PA (2 elementi) e QOL (5 elementi). Quando si risponde alle domande, si prende in considerazione la settimana precedente al giorno di compilazione del questionario. Le opzioni di risposta standardizzata sono fornite (5 caselle Likert) e ogni domanda ottiene un punteggio da 0 a 4, dove 0 indica nessun problema. I sei punteggi sono calcolati come la somma degli elementi inclusi, secondo i calcoli del punteggio dell' HOOS score. I punteggi grezzi vengono poi trasformati in una scala da 0 a 100, con zero che rappresenta problemi gravi di anca e/o inguine e 100 che rappresenta assenza di problemi di anca e/o inguine, come è comune nelle scale ortopediche.

Punteggi tra 0 e 100 rappresentano la percentuale del punteggio totale possibile raggiunto. Un punteggio cumulativo non viene calcolato poiché si ritiene opportuno analizzare e interpretare separatamente le diverse dimensioni.

HAGOS Foglio delle istruzioni manuali del punteggio

Assegna i seguenti punteggi alle caselle:

Nessuno Lieve Moderato Grave Estremo

0 1 2 3 4

Dati mancanti. Se un segno viene posizionato fuori da una casella, viene scelta la casella più vicina. Se ci sono due caselle segnate, è scelto ciò che indica i problemi più gravi. I dati mancanti sono trattati come tale; uno o due valori mancanti sono sostituiti con il valore medio per quella sottoscala. Se più di due elementi per le sottoscale PAIN, SYMPTOMS, ADL, SPORT/REC e QOL sono omessi, la risposta è considerata non valida e nessun punteggio è calcolato. Se viene omesso più di un valore per la sottoscala PA, la risposta è considerata

non valida e nessun punteggio è calcolato. Sommare il punteggio totale di ogni sottoscala e dividerlo per il possibile punteggio massimo per la scala. Tradizionalmente in ortopedia, 100 indica nessun problema e

0 indica problemi gravi. Il punteggio normalizzato viene trasformato per soddisfare questo standard.

Si prega di utilizzare le formule fornite per ogni sottoscala:

1. DOLORE $100 - \frac{Punteggio\ totale\ P1-P10\ x\ 100}{40} = 100 - \frac{\square}{40} = \frac{\square}{\square}$

2. SINTOMI $100 - \frac{Punteggio\ totale\ S1-S7\ x\ 100}{28} = 100 - \frac{\square}{28} = \frac{\square}{\square}$

3. ADL $100 - \frac{Punteggio\ totale\ A1-A5\ x\ 100}{20} = 100 - \frac{\square}{20} = \frac{\square}{\square}$

4. SPORT/REC $100 - \frac{Punteggio\ totale\ SP1-SP8\ x\ 100}{32} = 100 - \frac{\square}{32} = \frac{\square}{\square}$

5. PA $100 - \frac{Punteggio\ totale\ PA1-PA2\ x\ 100}{8} = 100 - \frac{\square}{8} = \frac{\square}{\square}$

6. QOL $100 - \frac{Punteggio\ totale\ Q1-Q5\ x\ 100}{20} = 100 - \frac{\square}{20} = \frac{\square}{\square}$

Profilo

Per visualizzare le differenze nelle sei diverse sottoscale di HAGOS e cambiare tra

diverse gestioni dell'HAGOS (ad es. dal pre-trattamento al post-trattamento), i profili HAGOS possono essere tracciati, come illustrato nell'esempio seguente.

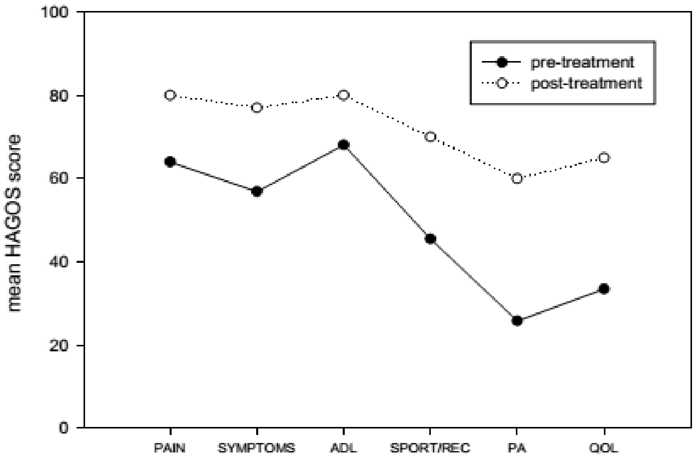

Supplement: Supplementary file 1 [file healthcare-12-01755-s001.zip › healthcare-3055745-supplementary.pdf]
